# Genetic predisposition for depression, psychosocial and behavioural factors and premenstrual symptoms: a cross-sectional study among young women in China

**DOI:** 10.1186/s12916-026-04913-w

**Published:** 2026-05-08

**Authors:** Yu Zhao, Qing Pan, Min Chen, Yanan Zhang, Grace W.K. Ho, Yifei Lin, Huan Song, Jin Huang, Yuchen Li, Donghao Lu

**Affiliations:** 1 Health Management Center, General Practice Medical Center, Innovation Institute for Integration of Medicine and Engineering, West China Hospital, Sichuan University, Chengdu, China; 2 West China School of Public Health and West China Fourth Hospital, Sichuan University, Chengdu, China; 3 Department of Anesthesiology and West China Biomedical Big Data Center, West China Hospital, Sichuan University, Chengdu, China; 4 School of Nursing, The Hong Kong Polytechnic University, Hong Kong, China; 5 Department of Urology, Lab of Health Data Science, Innovation Institute for Integration of Medicine and Engineering, West China Hospital, Sichuan University, Chengdu, China; 6 Mental Health Center, West China Hospital, Sichuan University, Chengdu, China; 7 Unit of Integrative Epidemiology, Institute of Environmental Medicine, Karolinska Institutet, Stockholm, Sweden

**Keywords:** Premenstrual symptoms, Premenstrual disorders, Depression, Genetic predisposition, Psychosocial and behavioural factors, Risk profile

## Abstract

**Background:**

Premenstrual disorders (PMDs) affect approximately one in three women of reproductive age and have a substantial impact on daily functioning and mental health. Given the challenges observed in diagnosis and clinical management, understanding whether genetic predisposition and readily assessable factors jointly mark greater symptom burden may help inform future risk-stratified monitoring and assessment.

**Methods:**

After excluding 424 participants with missing data, we conducted a cross-sectional study of 1528 female college students from the Care of Premenstrual Emotion (COPE) cohort in China. Premenstrual symptoms and probable PMD cases were assessed with the Calendar of Premenstrual Experiences. Four psychosocial and behavioural factors, including alcohol consumption, psychological resilience, adverse childhood experiences (ACEs), and body mass index (BMI), were recorded through electronic questionnaires. The polygenic risk score (PRS) of depression was derived from trans-ancestry genome-wide association study (GWAS) summary statistics. The associations and interactions of the PRS for depression and psychosocial and behavioural factors with premenstrual symptoms and probable PMD cases were examined.

**Results:**

The average age of the participants was 20.1 ± 1.59 years. Positive associations were observed between the depression PRS, alcohol consumption, low psychological resilience, ACEs, and premenstrual symptoms; additionally, positive associations between low psychological resilience, ACEs, and probable PMDs were observed. The psychosocial and behavioural factor score was associated with more severe premenstrual symptoms (*β* = 0.49, 95% CI: 0.35–0.62, *P* < 0.001) and higher odds of probable PMDs (OR = 1.97, 95% CI: 1.42–2.73, *P* < 0.001). Specifically, compared with participants with low depression PRS and no adverse psychosocial-behavioural factors, participants with high depression PRS and alcohol consumption (*β* = 0.32, 95% CI: 0.14–0.51, *P* = 0.001), low psychological resilience (*β* = 0.54, 95% CI: 0.33–0.75, *P* < 0.001) or ACEs (*β* = 0.26, 95% CI: 0.08–0.43, *P* = 0.003) exhibited more severe premenstrual symptoms; moreover, participants with high depression PRS and ≥ 2 psychosocial-behavioural factor scores demonstrated the greatest burden of premenstrual symptoms (*β* = 0.59, 95% CI: 0.37–0.81, *P* < 0.001) and higher odds of probable PMDs (OR = 1.79, 95% CI: 1.05–3.11, *P* = 0.035).

**Conclusions:**

If confirmed in prospective studies, a combined profile of genetic predisposition and psychosocial and behavioural factors may help identify young women who warrant closer evaluation for PMDs, and may inform future prevention-oriented studies focused on actionable exposures.

**Supplementary Information:**

The online version contains supplementary material available at 10.1186/s12916-026-04913-w.

## Background

Premenstrual disorders (PMDs), including premenstrual syndrome (PMS) and premenstrual dysphoric disorder (PMDD), are characterised by recurrent affective and somatic symptoms that emerge in the late luteal phase and remit soon after the onset of menses [1]. Although premenstrual symptoms are common, clinically significant PMDs are less frequent [1]. Epidemiological studies have reported that PMS affects approximately 20–30% of young adult women [2, 3], whereas PMDD (the more severe type of PMD) affects 2–6% of this population [1, 4]. PMDs are associated with impaired quality of life and interpersonal functioning and have been linked to psychiatric morbidity [1], perinatal depression [5], hypertension [6], and suicidal behaviour [5]. However, due to the complicated clinical diagnostic process (involving prospective symptom charting for at least two menstrual cycles), many affected women experience considerable delays in diagnosis and then treatment [7, 8]. Identifying women who may be at elevated risk before formal diagnosis could inform future risk-stratified approaches to prospective symptom monitoring and earlier clinical evaluation.

Current evidence suggests that PMDs are not caused by abnormal ovarian hormone concentrations per se, but by an abnormal sensitivity to normal cyclical hormonal fluctuations [9, 10]. However, it remains unclear as to what triggers these abnormal responses. Twin studies have reported substantial heritability of premenstrual symptoms, with estimates ranging from 35% to 57% [11–13]. Emerging data based on polygenic risk score (PRS) association analysis suggest that PMDs share genetic liability with a range of psychiatric disorders, including major depression, schizophrenia, and bipolar disorders [14]. These findings indicate that genetic predisposition may contribute to individual vulnerability to clinically significant premenstrual symptoms.

In parallel, psychosocial and behavioural factors represent important and potentially modifiable contributors to PMDs. Several psychosocial and behavioural factors have been identified for PMDs, including lower psychological resilience [15, 16], adverse childhood experiences (ACEs) [17, 18], alcohol consumption [19], smoking [20], and high body mass index (BMI) [21–23]. Because many of the psychosocial-behavioural factors often coexist in individuals, exploring the joint impact of these factors with PMDs is extremely relevant. Yet, there is very limited research on this topic. Moreover, existing research has largely examined genetic predisposition and environmental or behavioural factors separately. To our knowledge, no study has evaluated the joint and interactive associations of genetic predisposition and psychosocial-behavioural factors with premenstrual symptoms and probable PMDs.

Using a cohort of young women in China, the present study aimed to assess the associations of individual and composite psychosocial-behavioural factors with premenstrual symptoms and probable PMDs, as well as the joint and interactive associations of genetic predisposition and psychosocial-behavioural factors with these outcomes. By integrating genetic predisposition with psychosocial and behavioural factors, this study sought to provide preliminary evidence to identify young women who warrant prospective symptom monitoring and closer clinical evaluation before formal diagnosis, and to inform future prevention-oriented studies focusing on actionable factors related to PMDs.

## Methods

### Study population

This study was a cross-sectional analysis nested within the Care of Premenstrual Emotion (COPE), which is a prospective cohort study. The COPE cohort was established to collect information on demographic characteristics, health behaviours, social experience, digital biomarkers, and biospecimens of PMDs [24]. The recruited participants were female students at the Schools of Clinical Medicine, Stomatology, Basic Medicine and Forensic Medicine, Public Health, and Pharmacy from Sichuan University. All of the participants were fully informed about the study’s purpose and procedures and provided informed consent. Ethical approval for the COPE study was obtained from the Institutional Review Board of West China Hospital, Sichuan University (Approval No. 2018 − 535, 2022 − 1465 and 2023 − 179). From October 2021 to October 2024, a total of 2140 participants were enrolled and 1952 participants (91.2%) completed saliva sample collections by using an Oragene·DNA OG-510 self-collection kit (DNA Genotek Inc, Ottawa, Canada). After excluding 405 participants with missing data on psychosocial and behavioural factors and 19 participants who exhibited failure in genotype quality control, a total of 1528 participants were included in the final analysis.

### Genotyping and quality control

DNA was extracted from the saliva samples. Genotyping was performed on 1952 samples using the Infinium Chinese Genotyping Array v1.0, encompassing 727,394 single nucleotide polymorphisms (SNPs). Prequality control (QC) was then applied to both samples and variants. Samples exhibiting sex-genotype discordance were excluded. Variants with heterozygosity outliers (± 2 Standard deviations (SDs) from the mean), call rates less than 98%, or significant deviations from Hardy-Weinberg equilibrium (*P* < 1 × 10⁻⁶) were removed. Genotype imputation was performed by using the East Asian reference panel from the 1000 Genomes Project Phase 3, thus resulting in a total of 1951 samples and 10,159,805 SNPs. In the post-QC phase, variants with an imputation info score < 0.8, minor allele frequency (MAF) < 0.01, or cryptic relatedness (pairwise identity-by-descent > 0.25) were further removed. The final dataset included 1933 samples and 6,172,229 SNPs that passed all of the QC filters (Additional file 1: Fig. S1).

### Calculation of polygenic risk score

Due to the fact that PMDs share a substantial genetic architecture with major depressive disorder, schizophrenia, and bipolar disorder [14], the PRSs of these disorders were calculated. For major depressive disorder, we used summary statistics from the latest genome-wide association study (GWAS) meta-analysis by the Psychiatric Genomics Consortium, which included 537,363 cases and 2,061,567 controls of European, East Asian, South Asian, African, and Hispanic/Latin American descent [25]. Genome-wide significant SNPs (*P* < 5 × 10^− 8^) were selected, and linkage disequilibrium was accounted for via clumping of the target data by using an r² threshold of 0.1 within 250-kb windows. Each SNP was recoded as 0, 1, or 2 according to the number of risk alleles. The PRS was constructed by using the equation PRS = (β_1_ × SNP_1_ + β_2_ × SNP_2_ + … + β_n_ × SNP_n_)/*n*, where *n* is the total number of SNPs, and β denotes the effect size derived from the GWAS summary data [25]. The PRS was standardised as a z-score, and participants were stratified into tertiles (low, moderate, and high genetic risk) for association analyses. To evaluate the validity of the depression PRS, we examined the associations with depression symptoms and depression status by using linear and logistic regression analyses, respectively, adjusting for age and the first ten principal components of ancestry (Additional file 1: Table S1).

Similarly, the PRSs of schizophrenia and bipolar disorders were calculated based on the latest GWAS summary statistics [26, 27]. Due to the fact that no associations were observed between the PRSs of both schizophrenia and bipolar disorder and premenstrual symptoms or probable PMDs (Additional file 1: Table S2), our analyses focused on the PRS of depression.

### Assessment of psychosocial and behavioural factors

Based on prior knowledge [15, 17, 19, 21], four psychosocial and behavioural factors associated with PMDs were selected, including psychological resilience, ACEs, alcohol consumption, and BMI. Smoking was not included because of its low prevalence (1.1%) among young women in China [28]. Psychological resilience was measured by using the Chinese version of the Connor-Davidson Resilience Scale (CD-RISC) [29], which has demonstrated good reliability among Chinese individuals (Cronbach’s α coefficient = 0.91) [30]. The CD-RISC includes 25 items, scored on a 5-point Likert scale. Higher scores reflect a greater level of psychological resilience, and low psychological resilience was defined as the lowest quintile of the CD-RISC score. ACEs were assessed using the validated Chinese version of the Adverse Childhood Experiences International Questionnaire (ACE-IQ) [31]. This instrument includes 29 items evaluating 13 distinct ACE categories. The response formats varied by item and included dichotomous (yes/no), 5-point Likert scale, and 4-point Likert scale options. Exposure to each ACE category was recorded only when participants met the predefined minimum frequency threshold for the following adverse events within that domain: (1) physical abuse (required minimum frequency: many times), (2) emotional abuse (many times), (3) sexual abuse (ever), (4) physical neglect (many times), (5) emotional neglect (rarely or ever), (6) family violence (a few times or many times), (7) parental separation/divorce (yes), (8) household substance abuse (yes), (9) incarcerated household member (yes), (10) mental illness (yes), (11) community violence (many times), (12) bullying (many times), and (13) collective violence (ever). The status was recorded as exposed if participants endorsed at least 1 criterion among the 13 ACE categories. Alcohol consumption was assessed based on whether participants had consumed alcohol at least once in the past 30 days. BMI was calculated based on self-reported height and weight. Moreover, a composite psychosocial and behavioural factor score was calculated by summing the total number of these factors associated with premenstrual symptoms in our sample. Specifically, participants scored 1 point if they reported alcohol consumption, low resilience, or endorsement of any ACE. Due to the small sample size of the participants who were exposed to all three psychosocial and behavioural factors (4.6%), we combined those with a psychosocial and behavioural factor scores of 2 or 3 in the analysis.

### Assessment of premenstrual symptoms

At baseline, premenstrual symptoms, the primary outcome, were assessed by using a modified version of the Calendar of Premenstrual Experiences. The scale includes 8 emotional and 19 physical/behavioural symptom items, followed by 3 functional impact items. This scale exhibits a positive predictive value of 80% and has been validated by prospective daily symptom diaries [32]. Specifically, participants retrospectively rated symptoms experienced before menstruation over the past year. Each symptom was rated on a 4-point Likert scale (1 = none, 2 = mild, 3 = moderate, 4 = severe), and scores were summed to yield a total premenstrual symptom score (range: 27–108, Additional file 1: Table S3). The effects of premenstrual symptoms on daily activities and interpersonal relationships were also assessed.

As described in our previous study [24], the classification criteria for probable PMDs (the secondary outcome) were as follows: (1) the presence of at least one physical/behavioural symptom and at least one affective symptom; (2) moderate/severe overall symptom severity, moderate/severe impact on daily activities or interpersonal relationships, or more than one affective symptom rated as severe; (3) symptom onset occurring 1 to 14 days before menstruation; (4) symptom persistence for 1 to 7 days after the commencement of menstruation; and (5) complete resolution of symptoms within the first week following the cessation of menstruation.

### Covariates

Age [33, 34], academic school (including the Schools of Clinical Medicine, Stomatology, Basic Medicine and Forensic Medicine, Public Health, and Pharmacy) [34], and age at menarche [35] were self-reported at the baseline survey. Probable depression was assessed by using the Patient Health Questionnaire-9 (PHQ-9) with a cutoff score of 10 [36], and probable anxiety was assessed by using the Generalized Anxiety Disorder-7 (GAD-7) with a cutoff score of 10 [37]. The first ten principal components of ancestry were derived from the genotype data to account for population stratification [38]. All covariates had complete data.

### Statistical analysis

First, the mean and standard deviation of premenstrual symptoms, as well as the numbers and percentages of probable PMD cases, were summarised according to participant characteristics. Independent *t* tests and analysis of variance (ANOVA) were used to compare premenstrual symptoms across different characteristics, and the $\:{x}^{2}$ test was used to evaluate the differences in characteristics between participants with and without PMDs.

Afterwards, we individually examined the associations between psychosocial and behavioural factors and genetic predisposition. The associations of the PRS for depression with premenstrual symptoms were estimated by using linear regression models adjusted for age and the top ten principal components of ancestry. The associations of psychosocial and behavioural factors were adjusted for age, academic school, age at menarche, BMI, alcohol consumption, psychological resilience, and ACEs. Similar analyses for probable PMDs were performed by using logistic regression models.

To further investigate the joint effect of psychosocial and behavioural factors and genetic predisposition for depression, we examined the joint associations of PRS for depression and psychosocial and behavioural factors with premenstrual symptoms and probable PMDs. Moreover, we included interaction terms between these psychosocial and behavioural factors and the PRS for depression, with adjustments for age, school, age at menarche, BMI, alcohol consumption, psychological resilience, ACEs, and the first ten principal components of ancestry. The statistical significance of the interaction terms was evaluated using a likelihood ratio test, which compared the full model (including the interaction term) to a reduced model (excluding the interaction term). Such analyses were performed for both continuous premenstrual symptom scores and the binary outcome of probable PMDs.

To assess the robustness between the PRS for depression and premenstrual symptoms and probable PMDs, additional PRSs were constructed by using more liberal *P*-value thresholds (*P* < 1 × 10⁻⁶ and *P* < 1 × 10⁻⁵). To minimise the influence of potential population stratification, we performed a sensitivity analysis of the joint associations of PRS for depression and psychosocial and behavioural factors with both premenstrual symptoms and probable PMD cases while excluding outliers (± 3 SDs) along the first two ancestry principal components. As depression and anxiety are core components of premenstrual symptoms, to clarify whether psychosocial and behavioural factors independently contribute to premenstrual symptoms beyond these affective symptoms, we further conducted additional analyses with adjustment for probable depression and anxiety cases. Moreover, to further characterise symptom subtypes, we separately estimated these associations with affective symptoms and physical symptoms. However, due to the limited number of PMDD cases, comparative analyses between PMS and PMDD were not considered in the current analysis.

Statistical analyses were performed by using Plink (version 1.90) and R software (version 4.4.0), with a *P* value of less than or equal to 0.05 considered to indicate statistical significance.

## Results

### Characteristics

A total of 1528 female students with an average age of 20.1 ± 1.59 years were included; among them, 372 (24.3%) were assessed as probable PMD cases. Participants enrolled in schools other than Clinical Medicine, those with an earlier age at menarche, and those with comorbid depression or anxiety reported higher levels of premenstrual symptoms (Table 1). Similar, but less significant, trends were observed for probable PMDs.

**Table 1:** Comparisons of premenstrual symptoms and probable PMDs proportion across demographic and risk groups

Characteristics		Premenstrual symptoms		Probable PMDs	
	*N*	z-score, mean (SD)	*P*-value^1^	*N* (%)	*P*-value^2^
Age (years)			0.191		0.381
<=20	990	-0.02(1.00)		234(23.6)	
> 20	538	0.05(1.00)		138(25.7)	
Academic school			**0.029**		0.087
Clinical Medicine	1183	-0.03 (0.99)		276(23.3)	
Other^a^	345	0.11(1.04)		96(27.8)	
Age at menarche (years)			**0.017**		0.275
<12	350	0.13(1.07)		95(27.1)	
12–14	1111	-0.04(0.97)		264(23.8)	
>14	67	0.03(1.11)		13(19.4)	
Depression^b^			**< 0.001**		**0.001**
No	1401	-0.06(0.96)		326(23.3)	
Yes	127	0.66(1.17)		46(36.2)	
Anxiety^b^			**< 0.001**		**< 0.001**
No	1390	-0.07(0.95)		317(22.8)	
Yes	138	0.68(1.23)		55(39.9)	

Note: Abbreviation: SD, standard deviation. **a**: other biomedical schools: School of Stomatology, Basic Medical Sciences and Forensic Medicine, Public Health, and Pharmacy. **b**: Depression and Anxiety were assessed with the Patient Health Questionnaire-9 and Generalized Anxiety Disorder-7, both with a cutoff score of 10. ***P*** **value**^**1**^: Group comparison by premenstrual symptoms (z-score) (*t*-test/ANOVA); ***P*** **value**^**2**^: Group comparison by probable PMDs (chi-square test)

### Associations between the PRS for depression, psychosocial and behavioural factors and premenstrual symptoms

Compared with participants in the low PRS tertile, those in the high PRS tertile demonstrated significantly higher premenstrual symptom scores (*β* = 0.13, 95% CI: 0.00–0.25, Table 2). A borderline significant trend of increasing premenstrual symptoms across the PRS tertiles was also observed (*P* for trend = 0.05). All psychosocial and behavioural factors except BMI category, including alcohol consumption (*P* = 0.001), low psychological resilience (*P* < 0.001), and exposure to ACEs (*P* < 0.001), were positively associated with premenstrual symptoms.

**Table 2:** Associations of PRS for depression and psychosocial and behavioural factors with premenstrual symptoms and probable PMDs

Variables	*N*	Premenstrual symptoms			Probable PMDs		
		z-score, mean (SD)	β (95% CI)	*P*-value^1^	*N* (%)	OR (95% CI)	*P*-value^2^
**PRS of depression** ^**a**^							
Low PRS	504	-0.04(1.00)	Ref		113(22.4)	Ref	
Moderate PRS	520	0.00(0.99)	0.07 (-0.05, 0.20)	0.240	129(24.8)	1.18 (0.88, 1.58)	0.273
High PRS	504	0.04(1.01)	0.13 (0.00, 0.25)	**0.050**	130(25.8)	1.27 (0.95, 1.71)	0.112
* P* for trend				**0.050**			0.113
**Alcohol consumption** ^**b**^							
No	1099	-0.06(0.96)	Ref		252(22.9)	Ref	
Yes^d^	429	0.15(1.09)	0.19 (0.08, 0.30)	**0.001**	120(28.0)	1.26 (0.97, 1.62)	0.078
**Psychological resilience** ^**b**^							
**Quintiles**							
Q5	286	-0.14(0.99)	Ref		62(21.7)	Ref	
Q4	311	-0.06(0.95)	0.06(-0.10, 0.22)	0.462	70(22.5)	1.01(0.68, 1.49)	0.961
Q3	318	-0.09(0.89)	0.03(-0.13, 0.19)	0.702	67(21.1)	0.91(0.61, 1.35)	0.636
Q2	304	0.03(0.97)	0.14(-0.02, 0.30)	0.083	80(26.3)	1.23(0.83, 1.81)	0.301
Q1	309	0.27(1.14)	0.36(0.20, 0.52)	**< 0.001**	93(30.1)	1.45(0.99, 2.12)	0.056
P for trend				**< 0.001**			**0.026**
**Binary**							
High	1219	-0.07(0.95)	Ref		279(22.9)	Ref	
Low^e^	309	0.27(1.14)	0.30(0.18, 0.43)	**< 0.001**	93(30.1)	1.40(1.05, 1.85)	**0.020**
**ACEs** ^**b**^							
No	605	-0.14(0.94)	Ref		124(20.5)	Ref	
Yes	923	0.09(1.03)	0.21 (0.11, 0.31)	**< 0.001**	248(26.9)	1.38 (1.08, 1.78)	**0.011**
**BMI (kg/m** ^**2**^ **)** ^**b**^							
< 18.5	335	0.07(1.01)	0.11 (-0.02, 0.23)	0.090	80(23.9)	0.98 (0.73 to 1.30)	0.872
18.5–25	1108	-0.02(1.00)	Ref		278(25.1)	Ref	
≥ 25	85	-0.00(0.92)	0.00 (-0.22, 0.22)	0.981	14(16.5)	0.56 (0.30 to 0.98)	0.055
**PBS** ^**c**^							
0	384	-0.24(0.89)	Ref		73(19.0)	Ref	
1	697	-0.03(0.92)	0.20(0.08, 0.32)	**0.001**	157(22.5)	1.25(0.91, 1.71)	0.168
2–3	447	0.26(1.14)	0.49(0.35, 0.62)	**< 0.001**	142(31.8)	1.97(1.42, 2.73)	**< 0.001**
* P* for trend				**< 0.001**			**< 0.001**

Note: Abbreviation: SD, standard deviation; CI, confidence interval; OR, odds ratio; PRS, polygenic risk score; ACEs, adverse childhood experiences; BMI, body mass index. Premenstrual symptoms were standardised using z-score. PRS was standardised using z-scores, then categorised into low(< 33rd), moderate(33rd-67th) and high (> 67th) genetic risk groups. **a**: Age and the first ten principal components of ancestry were adjusted. **b**: Age, school, age at menarche, alcohol consumption, resilience, ACEs, and BMI were adjusted whenever applicable. **c**: PBS (psychosocial and behavioural factor score); Age, school and age at menarche were adjusted. **d**: Alcohol consumption was defined as consuming alcohol at least once during the past 30 days. **e**: Low psychological resilience: in the lowest quintile of the CD-RISC score

When the associated psychosocial and behavioural factors were analysed as a composite score, women with a score of 2–3 (i.e., women who were exposed to two or all three of the following factors: alcohol consumption, low psychological resilience, and ACEs) reported a greater burden of premenstrual symptoms (*β* = 0.49, 95% CI: 0.35–0.62) compared with unexposed women. A dose-response relationship was observed (*P* for trend < 0.001). Similarly, trends were observed for PMDs (*P* for trend < 0.001).

### Joint risk profile according to the depression PRS and psychosocial and behavioural factors

**Fig. 1:**
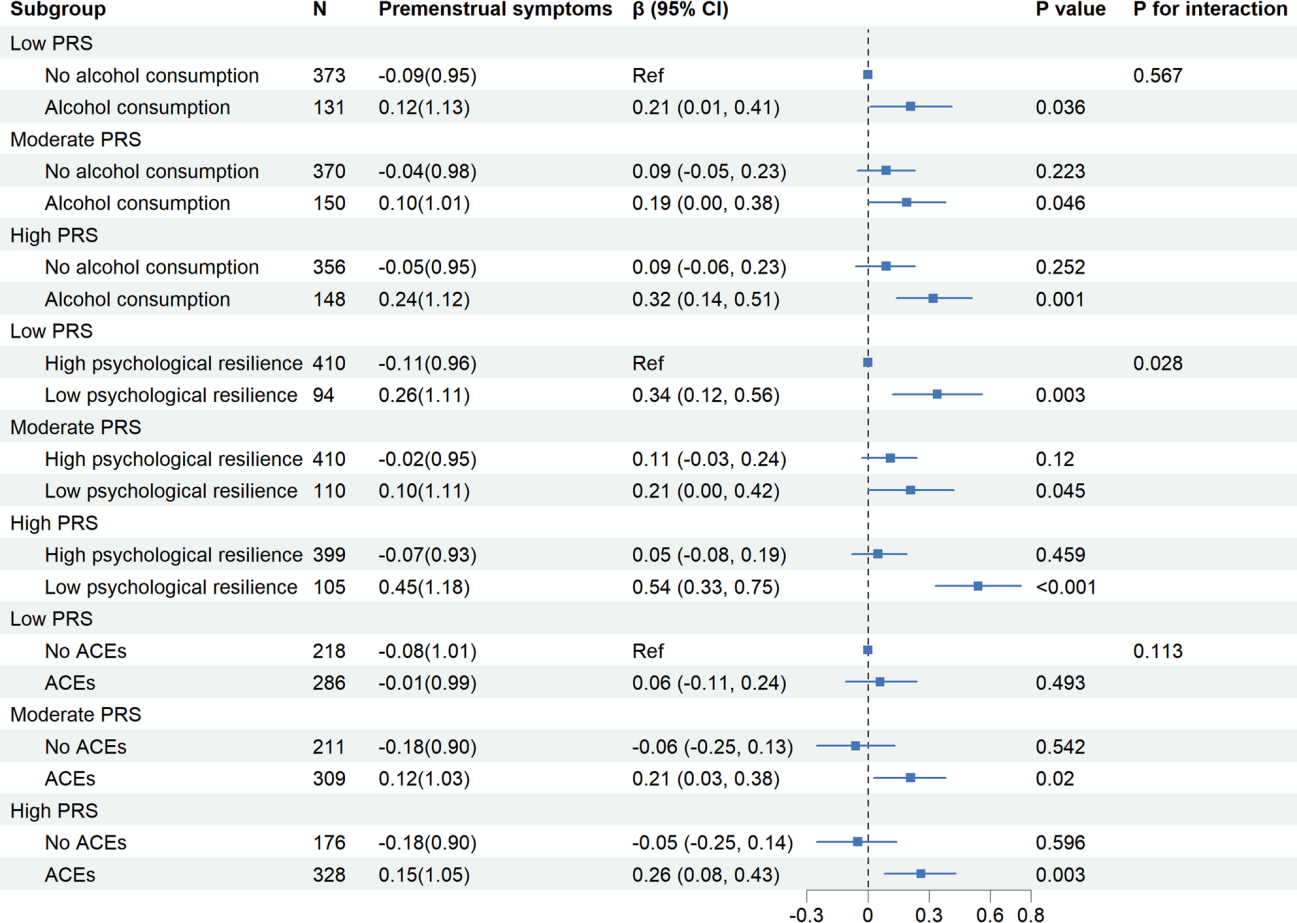
Joint associations of PRS for depression and psychosocial and behavioural factors with premenstrual symptoms. Note: Abbreviation: CI, confidence interval; PRS, polygenic risk score; ACEs, adverse childhood experiences. Age, school, age at menarche, BMI, first ten principal components of ancestry, alcohol consumption, resilience, and ACEs, whenever applicable, were adjusted. *P* for interaction was tested using a likelihood ratio test, comparing the full model (with interaction term) to the reduced model (without interaction term)

**Fig. 2:**
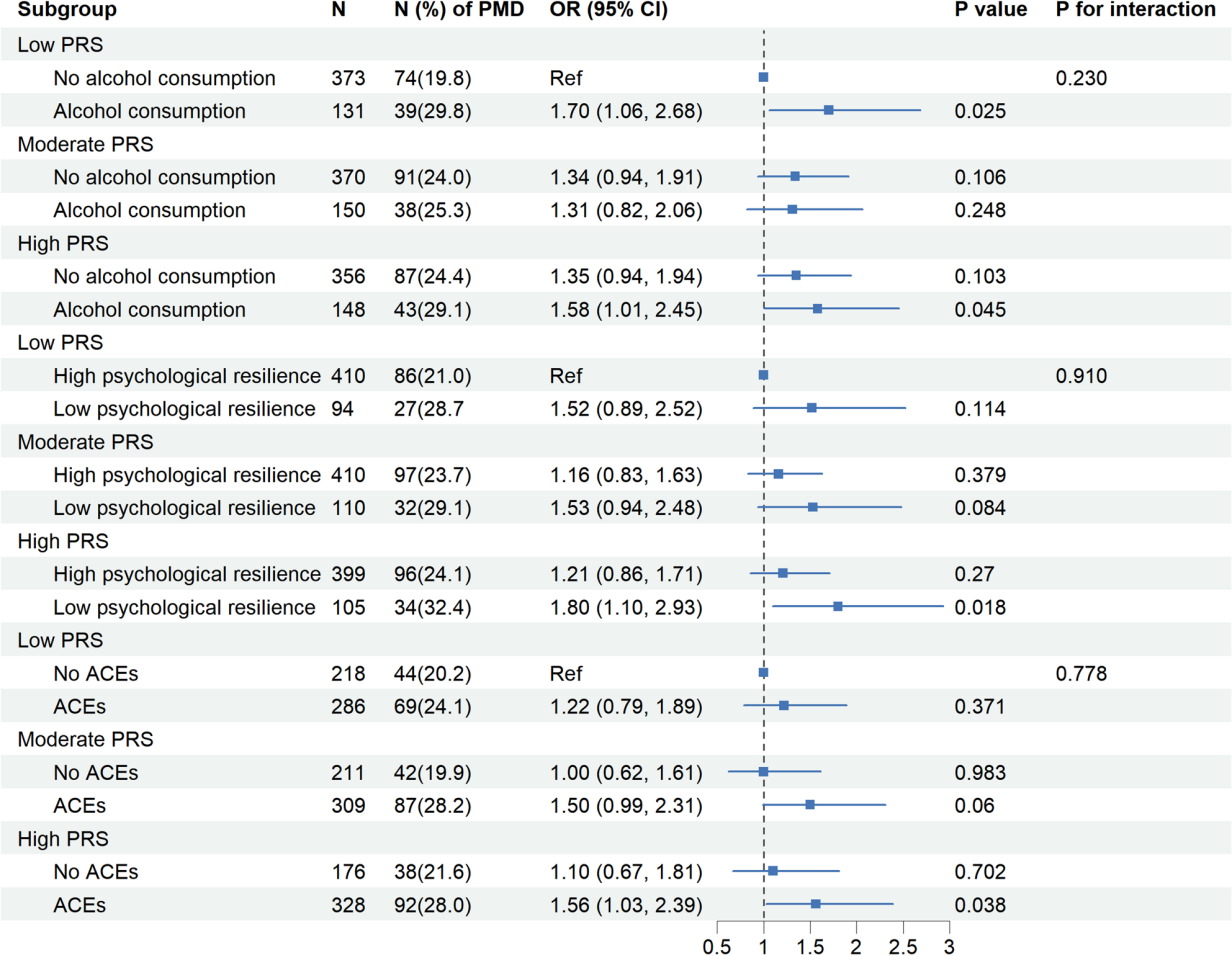
Joint associations of PRS for depression and psychosocial and behavioural factors with probable PMDs. Note: Abbreviation: OR, odds ratio; CI, confidence interval; PRS, polygenic risk score; ACEs, adverse childhood experiences. Age, school, age at menarche, alcohol consumption, resilience, ACEs, BMI and first ten principal components of ancestry were adjusted. *P* for interaction was tested using a likelihood ratio test, comparing the full model (with interaction term) to the reduced model (without interaction term)

When the associated psychosocial and behavioural factors were individually examined, women who were genetically predisposed to depression and who were exposed to adverse psychosocial and behavioural factors demonstrated significantly higher levels of premenstrual symptoms (e.g., high PRS and alcohol consumption: *β* = 0.32, 95% CI: 0.14–0.51, Fig. 1), compared with those with a low degree of genetic predisposition and no adverse psychosocial and behavioural factors. Similar patterns were observed for probable PMDs (Fig. 2).

**Fig. 3:**
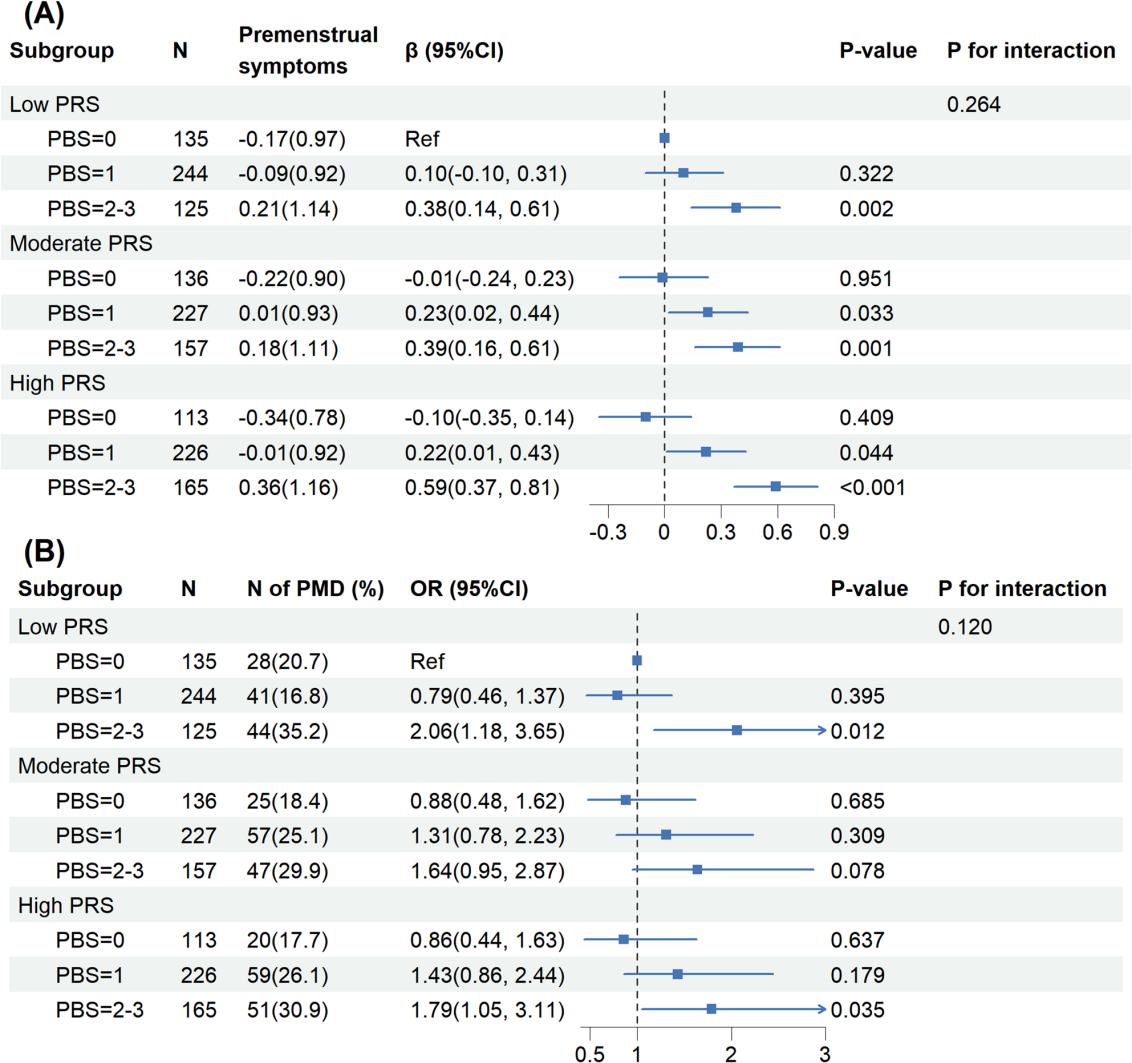
Joint associations of PRS for depression and psychosocial and behavioural factor score with premenstrual symptoms and probable PMDs. Note: (**A**) Premenstrual symptoms. (**B**) Probable PMDs. Abbreviation: CI, confidence interval; OR, odds ratio; PRS, polygenic risk score; PBS (psychosocial and behavioural factor score): participants scored 1 point if they had alcohol consumption, low psychological resilience, or exposure to ACEs. Age, school, age at menarche, BMI and the first ten principal components of ancestry were adjusted. *P* for interaction was tested using a likelihood ratio test, comparing the full model (with interaction term) to the reduced model (without interaction term)

When psychosocial and behavioural factors were analysed as a composite score, compared with women with low PRS and a psychosocial and behavioural factor score of 0, women who were genetically predisposed to depression and who had psychosocial and behavioural factor scores of 2–3 experienced the greatest burden of premenstrual symptoms (β = 0.59, 95% CI: 0.37–0.81, Fig. 3) and higher odds of probable PMDs (OR = 1.79, 95% CI: 1.05–3.11). Furthermore, a significant interaction effect between the depression PRS and psychological resilience on premenstrual symptoms was observed (*P* for interaction = 0.028).

### Additional analysis

Effect estimates were generally comparable across sensitivity analyses, including when alternative PRS thresholds were applied (Additional file 1: Table S4), after excluding ancestry outliers (Additional file 1: Table S5–S6), and after additionally adjusting for comorbid depression and anxiety (Additional file 1: Table S7–S8). When the symptomatology subtypes were analysed, similar patterns were noted between affective and physical premenstrual symptoms in terms of individual associations with the PRS for depression and psychosocial and behavioural factors (Additional file 1: Table S9), as well as joint effects (Additional file 1: Table S10–S11).

## Discussion

In the present study of 1528 young women in China, we demonstrated that the depression PRS and examined psychosocial and behavioural factors were individually associated with a greater burden of premenstrual symptoms, and some of these factors were also associated with higher odds of probable PMDs. Moreover, we demonstrated a potential risk profile by combining genetic susceptibility (using the PRS for depression as a proxy) with psychosocial and behavioural factor score. Specifically, compared with women with a low degree of genetic predisposition and a psychosocial and behavioural factor score of 0, women with a high degree of genetic predisposition and psychosocial and behavioural factor scores of 2–3 experienced a much greater burden of premenstrual symptoms and higher odds of probable PMDs. These findings suggest that a combined risk profile, integrating genetic predisposition with readily assessable psychosocial and behavioural factors, may help identify young women who warrant prospective symptom monitoring and closer clinical evaluation before formal diagnosis, and may inform future prevention-oriented studies focused on actionable exposures.

In a previous study involving 56,725 women of European ancestry, PRSs of psychiatric disorders (particularly including major depression, schizophrenia, and bipolar disorder) [14] were observed to be associated with symptoms of PMDs, as measured by using two screening questions. Aligning with these results, our study provided first-hand evidence in an East Asian population that women with a higher PRS for depression exhibited more severe premenstrual symptoms, as comprehensively assessed using 27 symptom items. Affective symptoms are a cardinal component of PMD symptomatology and constitute a key dimension of their diagnostic framework. Moreover, women with PMDs exhibit high comorbidity with major depression [39]. However, this scenario cannot completely explain our findings given that comparable results were obtained after we adjusted for depression. Although it is not implausible that depression-related genetic liability is biologically relevant to PMDs, future research based on PRSs for PMDs is warranted. In addition, our study revealed no associations of the PRSs for schizophrenia and bipolar disorder with PMDs. The absence of associations for schizophrenia and bipolar disorder may be explained by both the limited GWAS sample sizes for these conditions and their low prevalence reported in China and in our study [40, 41], thus suggesting minimal underlying genetic risk in this population.

Regarding known psychosocial and behavioural factors, our findings are consistent with the results of previous studies involving alcohol consumption [19], low psychological resilience [15], and exposure to ACEs [17]. However, no significant association was observed for BMI categories with premenstrual symptoms or the risk of PMDs, which may be attributable to the relatively low prevalence of overweight (5.6%) in our study population. More importantly, although previous studies have predominantly examined individual psychosocial and behavioural factors, few have explored their cumulative effects. Our study contributes to this knowledge by demonstrating that a greater number of adverse exposures is associated with more severe premenstrual symptoms and a higher risk of PMDs in a dose-response manner.

Identifying high-risk profiles may provide preliminary evidence to inform future efforts to improve the detection and targeted prevention of PMDs in clinical and public health settings. A combined profile based on genetic predisposition and readily assessable factors may help identify young women who warrant more detailed assessment and follow-up. To the best of our knowledge, this is the first study to demonstrate the differential risk across subgroups by integrating genetic predisposition and psychosocial and behavioural factors for premenstrual symptoms and PMDs. Premenstrual symptoms appeared to increase with increasing genetic susceptibility and psychosocial and behavioural factor scores; additionally, a significant interaction between the depression PRS and psychological resilience in relation to premenstrual symptoms was observed. Notably, the influence of psychosocial and behavioural factors on PMDs risk seemed to be stronger than the PRS for depression, given the observation of comparable ORs of PMDs associated with psychosocial and behavioural factor scores of 2–3 across the PRS tertiles. However, future studies with larger sample sizes are needed to determine gene and environmental interactions.

The strength of our study is based on the use of diverse data including both genotype and phenotypic information, as well as the use of a validated scale for assessing premenstrual symptoms and probable PMD diagnosis. However, our study has several limitations. First, the cross-sectional nature of our analysis of alcohol consumption, psychological resilience and BMI limits the ability to establish temporal relationships with premenstrual symptoms or PMD status. However, genetic susceptibility to depression and the occurrence of ACEs are unlikely to be influenced by reverse causation. Second, we used trans-ancestry GWAS summary statistics to calculate the depression PRS in East Asian populations, and potential differences in genetic architecture may affect predictive accuracy [42, 43]. Although several GWASs of major depressive disorder have been performed in East Asian cohorts, the relatively small sample size poses limitations. In our sample, PRSs derived from East Asian GWASs demonstrated limited risk stratification capacity and no significant association with depression. Future studies using large GWAS datasets from ancestrally matched populations are warranted to improve PRS accuracy and to validate our findings. Third, misclassification bias could arise from self-reported data, such as BMI calculated from self-reported height and weight, which may lead to underestimation of true values due to reporting errors [44]. Fourth, although we adjusted for key potential confounders (such as age and age at menarche), residual confounding factors may persist due to unmeasured variables, such as smoking, although its prevalence is expected to be very low (1.1%) among female college students in China [28]. Fifth, potential selection bias may arise from the exclusion of participants with missing data. However, the overall similarity in participant characteristics and the incorporation of relevant covariates in the analyses are expected to reduce its impact (Additional file 1: Table S12). Finally, our study population consisted of young Chinese female medical students with a relatively narrow age range, which may hinder the generalisability of our findings to other populations or age groups. However, the prevalence of PMDs in our study is consistent with that in other reports from population-based studies [4], although the PMD cases were not confirmed by prospective symptom charting. Due to the fact that the prevalence of PMDs increases with age across the reproductive lifespan [3, 45, 46], severe cases may be underrepresented in our sample. Future studies adopting comparable designs in older and more representative populations (such as recruitment at ages 30 or 40) would be valuable to assess the persistence, progression, and generalisability of these findings and to better capture more severe PMDs.

## Conclusions

In the present study of young women in China, both the genetic predisposition to depression and psychosocial and behavioural factors (such as alcohol consumption, low psychological resilience and ACEs) were observed to be individually and jointly associated with premenstrual symptoms, and some of these factors were associated with higher odds of probable PMDs. If confirmed in prospective studies, a combined risk profile based on genetic predisposition and readily assessable psychosocial and behavioural factors may help identify young women who warrant prospective symptom monitoring and closer clinical evaluation. These findings may also inform future studies evaluating whether psychosocial and behavioural factors associated with PMDs could serve as potential targets for reducing symptom burden, particularly among individuals with high genetic predisposition for depression.

## Supplementary Information

Below is the link to the electronic supplementary material.

Supplementary Material 1: Supplementary Tables S1–S12 and Figure S1.

## Data Availability

The de-identified datasets used during the current study are available from the corresponding author upon reasonable request.

## Abbreviations

ACE-IQ: Adverse Childhood Experiences International Questionnaire
ACEs: Adverse childhood experiences
ANOVA: Analysis of variance
BMI: Body mass index
CD-RISC: Connor-Davidson Resilience Scale
CI: Confidence interval
COPE: Care of Premenstrual Emotion
DNA: Deoxyribonucleic acid
GAD-7: Generalized Anxiety Disorder-7
GWAS: Genome-wide association study
MAF: Minor allele frequency
OR: Odds ratio
PBS: Psychosocial and behavioural factor score
PHQ-9: Patient Health Questionnaire-9
PMDD: Premenstrual dysphoric disorder
PMDs: Premenstrual disorders
PMS: Premenstrual syndrome
PRS: Polygenic risk score
QC: Quality control
SDs: Standard deviations
SNPs: Single nucleotide polymorphisms
