# Supplementary material for: Genetic predisposition for depression, psychosocial and behavioural factors and premenstrual symptoms: a cross-sectional study among young women in China
Source: BMC Med. 2026 May 8;24:371. doi: 10.1186/s12916-026-04913-w (PMC13325797; doi:10.1186/s12916-026-04913-w)
Supplement: Supplementary file 1 — Supplementary Material 1: Supplementary Tables S1–S12 and Figure S1. [file 12916_2026_4913_MOESM1_ESM.docx]

[Table S1. Associations of PRS for depression with depression symptoms and probable depression cases 2](#_Toc229147998)

[Table S2. Associations of PRS for schizophrenia/bipolar disorder with premenstrual symptoms and probable PMDs 2](#_Toc229147999)

[Table S3. Calendar of Premenstrual Experiences Scale 3](#_Toc229148000)

[Table S4. Associations between PRS for depression and premenstrual symptoms and probable PMDs 4](#_Toc229148001)

[Table S5. Joint associations of PRS for depression and psychosocial and behavioural factors with premenstrual symptoms and probable PMDs after excluding ancestry outliers 5](#_Toc229148002)

[Table S6. Joint associations of PRS for depression and psychosocial and behavioural factor score with premenstrual symptoms and probable PMDs after excluding ancestry outliers 7](#_Toc229148003)

[Table S7. Joint associations of PRS for depression and psychosocial and behavioural factors with premenstrual symptoms and probable PMDs, additionally adjusted for depression and anxiety 8](#_Toc229148004)

[Table S8. Joint associations of PRS for depression and psychosocial and behavioural factor score with premenstrual symptoms and probable PMDs, additionally adjusted for depression and anxiety 10](#_Toc229148005)

[Table S9. Associations of PRS for depression and psychosocial and behavioural factors with premenstrual affective and physical symptoms 10](#_Toc229148006)

[Table S10. Joint associations of PRS for depression and psychosocial and behavioural factors with premenstrual affective/physical symptoms 12](#_Toc229148007)

[Table S11. Joint associations of PRS for depression and psychosocial and behavioural factor score with premenstrual affective/physical symptoms 13](#_Toc229148008)

[Table S12. Comparison of characteristics between included and excluded participants 15](#_Toc229148009)

[Figure S1. The quality control and imputation procedure of COPE 16](#_Toc229148010)

## Table S1. Associations of PRS for depression with depression symptoms and probable depression cases

| **PRS of depression** | ***N*** | **Depression symptoms** | | | **Probable depression cases** | | |
| --- | --- | --- | --- | --- | --- | --- | --- |
|  |  | **z-score (mean [SD])** | ***β* (95%CI)** | ***P* value^1^** | **N (%)** | **OR (95%CI)** | ***P* value^2^** |
| Low PRS | 504 | -0.07(0.97) | Ref |  | 34(6.7) | Ref |  |
| Moderate PRS | 520 | 0.00(0.98) | 0.09(-0.03 to 0.22) | 0.151 | 44(8.5) | 1.45(0.90 to 2.41) | 0.128 |
| High PRS | 504 | 0.07(1.05) | 0.15(0.03 to 0.28) | **0.017** | 49(9.7) | 1.54(0.97 to 2.57) | 0.075 |
| *P* for trend |  |  |  | **0.017** |  |  | 0.081 |

**Note:** The PRS was standardised using z-score, then categorized into low(<33rd), moderate(33rd-67th) and high (>67th) genetic risk groups. Multiple linear and logistic regression models were used to estimate the associations between PRS of depression and depression symptoms and cases, respectively. The first ten principal components of ancestry and age were adjusted in the analysis.

## Table S2. Associations of PRS for schizophrenia/bipolar disorder with premenstrual symptoms and probable PMDs

|  | ***N*** | **Premenstrual symptoms** | | | **Probable PMDs** | | |
| --- | --- | --- | --- | --- | --- | --- | --- |
|  |  | **z-score (mean [SD])** | ***β* (95%CI)** | ***P* value^1^** | **N (%)** | **OR (95%CI)** | ***P* value^2^** |
| **PRS of schizophrenia** |  |  |  |  |  |  |  |
| Low PRS | 504 | 0.02(0.99) | Ref |  | 123(24.4) | Ref |  |
| Moderate PRS | 520 | 0.01(1.03) | -0.02(-0.14 to 0.11) | 0.791 | 130(25.0) | 1.04(0.77 to 1.39) | 0.809 |
| High PRS | 504 | -0.02(0.98) | -0.05(-0.18 to 0.08) | 0.440 | 119(23.6) | 0.96(0.71 to 1.30) | 0.796 |
| *P* for trend |  |  |  | 0.438 |  |  | 0.790 |
| **PRS of bipolar disorder** |  |  |  |  |  |  |  |
| Low PRS | 504 | 0.01(1.02) | Ref |  | 121(24.0) | Ref |  |
| Moderate PRS | 520 | -0.01(1.00) | -0.00(-0.13 to 0.12) | 0.943 | 125(24.0) | 1.02(0.76 to 1.37) | 0.887 |
| High PRS | 504 | -0.01(0.99) | -0.01(-0.13 to 0.12) | 0.912 | 126(25.0) | 1.08(0.80 to 1.45) | 0.625 |
| *P* for trend |  |  |  | 0.912 |  |  | 0.624 |

**Note:** The PRS was standardised using z-score, then categorized into low(<33rd), moderate(33rd-67th) and high (>67th) genetic risk groups. Multiple linear and logistic regression models were used to estimate the associations between PRS of schizophrenia/bipolar disorder and premenstrual symptoms and probable PMDs, respectively. The first ten principal components of ancestry and age were adjusted in the analysis

## Table S3. Calendar of Premenstrual Experiences Scale

| **Symptoms** | **Severity** | | | |
| --- | --- | --- | --- | --- |
|  | **None** | **Mild** | **Moderate** | **Severe** |
| 1 Abdominal bloating |  |  |  |  |
| 2 Breast tenderness |  |  |  |  |
| 3 Dizziness |  |  |  |  |
| 4 Headache |  |  |  |  |
| 5 Hot flashes |  |  |  |  |
| 6 Nausea |  |  |  |  |
| 7 Swelling in the extremities |  |  |  |  |
| 8 Acne |  |  |  |  |
| 9 Diarrhea |  |  |  |  |
| 10 Food cravings |  |  |  |  |
| 11 Palpitations |  |  |  |  |
| 12 Anxiety |  |  |  |  |
| 13 Increased appetite |  |  |  |  |
| 14 Decreased appetite |  |  |  |  |
| 15 Irritability |  |  |  |  |
| 16 Emotional hypersensitivity |  |  |  |  |
| 17 Fatigue |  |  |  |  |
| 18 Mood swings |  |  |  |  |
| 19 Tearfulness |  |  |  |  |
| 20 Insomnia |  |  |  |  |
| - 21 Angry outbursts |  |  |  |  |
| 22 Increased desire to be alone |  |  |  |  |
| 23 Depression |  |  |  |  |
| 24 Confusion |  |  |  |  |
| 25 Forgetfulness |  |  |  |  |
| 26 Abdominal cramping |  |  |  |  |
| 27 Lower back pain |  |  |  |  |

**Note:** PMD symptoms were classified as none, mild, moderate and severe, which were coded as 1, 2, 3 and 4, respectively. Affective symptoms include 12, 15, 16, 18, 19, 21, 22, 23; Physical symptoms include 1, 2, 3, 4, 5, 6, 7, 8, 9, 10, 11, 13, 14, 17, 20, 24, 25, 26, 27.

## Table S4. Associations between PRS for depression and premenstrual symptoms and probable PMDs

|  | ***N*** | **Premenstrual symptoms** | | | **Probable PMDs** | | |
| --- | --- | --- | --- | --- | --- | --- | --- |
|  |  | **z-score, mean (SD)** | ***β* (95%CI)** | ***P* value^1^** | **N (%)** | ***OR* (95%CI)** | ***P* value^2^** |
| **PRS of depression(1e-6)** |  |  |  |  |  |  |  |
| Low PRS | 504 | -0.09(0.97) | Ref |  | 112(22.2) | Ref |  |
| Moderate PRS | 520 | 0.04(0.97) | 0.18(0.05, 0.30) | **0.005** | 140(26.9) | 1.37(1.03, 1.84) | **0.033** |
| High PRS | 504 | 0.05(1.05) | 0.19(0.06, 0.31) | **0.003** | 120(23.8) | 1.16(0.86, 1.58) | 0.325 |
| P for trend | | | | **0.017** |  |  | 0.329 |
| **PRS of depression(1e-5)** |  |  |  |  |  |  |  |
| Low PRS | 504 | -0.02(0.99) | Ref |  | 120(23.8) | Ref |  |
| Moderate PRS | 520 | 0.02(1.00) | 0.10(-0.02, 0.23) | 0.099 | 130(25.0) | 1.13(0.85, 1.52) | 0.407 |
| High PRS | 504 | 0.00(1.01) | 0.08(-0.04, 0.31) | 0.203 | 122(24.2) | 1.09(0.81, 1.47) | 0.566 |
| P for trend | | | | 0.198 |  |  | 0.569 |

**Note:** The PRS was standardised using z-score, then categorized into low(<33rd), moderate(33rd-67th) and high (>67th) genetic risk groups. Multiple linear and logistic regression models were used to estimate the associations between the PRS of depression and premenstrual symptoms and PMDs cases, respectively. The first ten principal components of ancestry and age were adjusted in the analysis.

## Table S5. Joint associations of PRS for depression and psychosocial and behavioural factors with premenstrual symptoms and probable PMDs after excluding ancestry outliers

|  | ***N*** | **Premenstrual symptoms**  **(z-score)** | ***β*** | **95%CI** | ***P* value^1^** | **N (%) of PMD** | ***OR*** | **95%CI** | ***P* value^2^** |
| --- | --- | --- | --- | --- | --- | --- | --- | --- | --- |
| **Low PRS** |  |  |  |  |  |  |  |  |  |
| No alcohol consumption | 360 | -0.11(0.94) | Ref |  |  | 83(23.1) | Ref |  |  |
| Alcohol consumption | 124 | 0.01(1.05) | 0.08 | -0.12 to 0.29 | 0.412 | 35(28.2) | 1.26 | 0.78 to 2.00 | 0.335 |
| **Moderate PRS** |  |  |  |  |  |  |  |  |  |
| No alcohol consumption | 352 | 0.01(0.95) | 0.13 | -0.02 to 0.28 | 0.082 | 80(22.7) | 0.97 | 0.68 to 1.38 | 0.845 |
| Alcohol consumption | 146 | 0.22(1.04) | 0.31 | 0.12 to 0.50 | **0.001** | 43(29.5) | 1.37 | 0.88 to 2.12 | 0.160 |
| **High PRS** |  |  |  |  |  |  |  |  |  |
| No alcohol consumption | 344 | -0.08(0.99) | 0.05 | -0.10 to 0.19 | 0.535 | 77(22.4) | 0.96 | 0.67 to 1.38 | 0.846 |
| Alcohol consumption | 140 | 0.20(1.15) | 0.30 | 0.10 to 0.49 | **0.003** | 33(23.6) | 0.96 | 0.59 to 1.52 | 0.856 |
| ***P* for interaction** |  |  |  |  | 0.513 |  |  |  | 0.544 |
| **Low PRS** |  |  |  |  |  |  |  |  |  |
| High psychological resilience | 392 | -0.14(0.93) | Ref |  |  | 91(23.2) | Ref |  |  |
| Low psychological resilience | 92 | 0.18(1.10) | 0.30 | 0.08 to 0.53 | 0.008 | 27(29.3) | 1.36 | 0.80 to 2.26 | 0.246 |
| **Moderate PRS** |  |  |  |  |  |  |  |  |  |
| High psychological resilience | 382 | 0.02(0.94) | 0.17 | 0.03 to 0.31 | 0.017 | 86(22.5) | 0.95 | 0.68 to 1.34 | 0.785 |
| Low psychological resilience | 116 | 0.24(1.10) | 0.35 | 0.15 to 0.56 | **0.001** | 37(31.9) | 1.51 | 0.94 to 2.39 | 0.086 |
| **High PRS** |  |  |  |  |  |  |  |  |  |
| High psychological resilience | 398 | -0.08(0.98) | 0.07 | -0.07 to 0.21 | 0.317 | 85(21.4) | 0.89 | 0.63 to 1.26 | 0.509 |
| Low psychological resilience | 86 | 0.41(1.22) | 0.53 | 0.30 to 0.77 | **<0.001** | 25(29.1) | 1.32 | 0.76 to 2.24 | 0.313 |
| ***P* for interaction** |  |  |  |  | 0.205 |  |  |  | 0.912 |
| **Low PRS** |  |  |  |  |  |  |  |  |  |
| No ACEs | 222 | -0.11(0.99) | Ref |  |  | 53(23.9) | Ref |  |  |
| ACEs | 262 | -0.05(0.95) | 0.03 | -0.15 to 0.21 | 0.744 | 65(24.8) | 1.00 | 0.65 to 1.53 | 0.997 |
| **Moderate PRS** |  |  |  |  |  |  |  |  |  |
| No ACEs | 184 | -0.16(0.88) | -0.02 | -0.22 to 0.17 | 0.827 | 33(17.9) | 0.69 | 0.42 to 1.13 | 0.140 |
| ACEs | 314 | 0.21(1.01) | 0.31 | 0.14 to 0.48 | **0.001** | 90(28.7) | 1.26 | 0.85 to 1.90 | 0.252 |
| **High PRS** |  |  |  |  |  |  |  |  |  |
| No ACEs | 180 | -0.17(0.91) | -0.05 | -0.24 to 0.15 | 0.637 | 32(17.8) | 0.68 | 0.41 to 1.12 | 0.134 |
| ACEs | 304 | 0.11(1.10) | 0.23 | 0.05 to 0.40 | **0.011** | 78(25.7) | 1.08 | 0.72 to 1.64 | 0.718 |
| ***P* for interaction** |  |  |  |  | 0.060 |  |  |  | 0.132 |

**Note:** PRS was standardised using z-scores, then categorized into low(<33rd), moderate(33rd-67th) and high (>67th) genetic risk groups. Premenstrual symptoms were standardised using z-score. Low psychological resilience was defined as the lowest quintile of CD-RISC scale. Age, school, age at menarche, alcohol consumption, resilience, ACEs, BMI, and first ten principal components of ancestry were adjusted. *P* for interaction was tested using a likelihood ratio test, comparing the full model (with interaction term) to the reduced model (without interaction term).

## Table S6. Joint associations of PRS for depression and psychosocial and behavioural factor score with premenstrual symptoms and probable PMDs after excluding ancestry outliers

| **Subgroup** | ***N*** | **Premenstrual symptoms** | | | **probable PMDs** | | |
| --- | --- | --- | --- | --- | --- | --- | --- |
|  |  | **z-score, mean (SD)** | ***β* (95%CI)** | ***P* value^1^** | **N (%)** | ***OR* (95%CI)** | ***P* value^2^** |
| **Low PRS** |  |  |  |  |  |  |  |
| PBS=0 | 141 | -0.19(0.95) | Ref |  | 32(22.7) | Ref |  |
| PBS=1 | 224 | -0.10(0.89) | 0.07(-0.14, 0.28) | 0.490 | 48(21.4) | 0.91(0.55, 1.54) | 0.732 |
| PBS=2-3 | 119 | 0.10(1.11) | 0.27(0.03, 0.51) | **0.027** | 38(31.9) | 1.55(0.89, 2.72) | 0.126 |
| **Moderate PRS** |  |  |  |  |  |  |  |
| PBS=0 | 111 | -0.28(0.79) | -0.07(-0.32, 0.18) | 0.580 | 15(13.5) | 0.52(0.26, 1.02) | 0.062 |
| PBS=1 | 232 | 0.07(0.93) | 0.28(0.07, 0.48) | **0.008** | 56(24.1) | 1.09(0.66, 1.81) | 0.736 |
| PBS=2-3 | 155 | 0.34(1.10) | 0.53(0.30, 0.76) | **<0.001** | 52(33.5) | 1.76(1.04, 3.00) | **0.035** |
| **High PRS** |  |  |  |  |  |  |  |
| PBS=0 | 120 | -0.28(0.86) | -0.07(-0.31, 0.17) | 0.569 | 22(18.3) | 0.77(0.41, 1.41) | 0.401 |
| PBS=1 | 213 | -0.05(0.95) | 0.16(-0.05, 0.37) | 0.147 | 46(21.6) | 0.95(0.57, 1.61) | 0.843 |
| PBS=2-3 | 151 | 0.31(1.22) | 0.53(0.30, 0.76) | **<0.001** | 42(27.8) | 1.31(0.76, 2.26) | 0.334 |
| ***P* for interaction** |  | 0.209 | | | 0.283 | | |

**Note: PBS** (psychosocial and behavioural factor score): participants scored 1 point if they had alcohol consumption, low resilience or exposure to ACEs. PRS was standardised using z-scores, then categorized into low(<33rd), moderate(33rd-67th) and high (>67th) genetic risk groups. Premenstrual symptoms were standardized using z-score. Low psychological resilience was defined as the lowest quintile of CD-RISC scale. Age, school, age at menarche, BMI, and the first ten principal components of ancestry were adjusted. *P* for interaction was tested using a likelihood ratio test, comparing the full model (with interaction term) to the reduced model (without interaction term).

## Table S7. Joint associations of PRS for depression and psychosocial and behavioural factors with premenstrual symptoms and probable PMDs, additionally adjusted for depression and anxiety

|  | ***N*** | **Premenstrual symptoms**  **(z-score)** | ***β*** | **95%CI** | ***P* value** | **N (%) of PMD** | ***OR*** | **95%CI** | ***P* value** |
| --- | --- | --- | --- | --- | --- | --- | --- | --- | --- |
| **Low PRS** |  |  |  |  |  |  |  |  |  |
| No alcohol consumption | 373 | -0.09(0.95) | Ref |  |  | 74(19.8) | Ref |  |  |
| Alcohol consumption | 131 | 0.12(1.13) | 0.20 | 0.01 to 0.40 | **0.037** | 39(29.8) | 1.69 | 1.06 to 2.67 | **0.027** |
| **Moderate PRS** |  |  |  |  |  |  |  |  |  |
| No alcohol consumption | 370 | -0.04(0.98) | 0.08 | -0.06 to 0.21 | 0.286 | 91(24.0) | 1.32 | 0.93 to 1.89 | 0.127 |
| Alcohol consumption | 150 | 0.10(1.01) | 0.13 | -0.06 to 0.31 | 0.173 | 38(25.3) | 1.22 | 0.77 to 1.93 | 0.393 |
| **High PRS** |  |  |  |  |  |  |  |  |  |
| No alcohol consumption | 356 | -0.05(0.95) | 0.07 | -0.08 to 0.21 | 0.364 | 87(24.4) | 1.32 | 0.92 to 1.91 | 0.130 |
| Alcohol consumption | 148 | 0.24(1.12) | 0.30 | 0.11 to 0.48 | **0.002** | 43(29.1) | 1.55 | 0.98 to 2.42 | 0.057 |
| ***P* for interaction** |  |  |  |  | 0.351 |  |  |  | 0.187 |
| **Low PRS** |  |  |  |  |  |  |  |  |  |
| High psychological resilience | 410 | -0.11(0.96) | Ref |  |  | 86(21.0) | Ref |  |  |
| Low psychological resilience | 94 | 0.26(1.11) | 0.20 | -0.02 to 0.41 | 0.079 | 27(28.7 | 1.33 | 0.77 to 2.24 | 0.295 |
| **Moderate PRS** |  |  |  |  |  |  |  |  |  |
| High psychological resilience | 410 | -0.02(0.95) | 0.07 | -0.06 to 0.20 | 0.296 | 97(23.7) | 1.13 | 0.80 to 1.58 | 0.791 |
| Low psychological resilience | 110 | 0.10(1.11) | 0.08 | -0.12 to 0.29 | 0.437 | 32(29.1) | 1.36 | 0.82 to 2.22 | 0.224 |
| **High PRS** |  |  |  |  |  |  |  |  |  |
| High psychological resilience | 399 | -0.07(0.93) | 0.05 | -0.09 to 0.18 | 0.504 | 96(24.1) | 1.19 | 0.85 to 1.68 | 0.308 |
| Low psychological resilience | 105 | 0.45(1.18) | 0.39 | 0.18 to 0.91 | **<0.001** | 34(32.4) | 1.54 | 0.93 to 2.55 | 0.093 |
| ***P* for interaction** |  |  |  |  | 0.071 |  |  |  | 0.963 |
| **Low PRS** |  |  |  |  |  |  |  |  |  |
| No ACEs | 218 | -0.08(1.01) | Ref |  |  | 44(20.2) | Ref |  |  |
| ACEs | 286 | -0.01(0.99) | 0.03 | -0.14 to 0.20 | 0.695 | 69(24.1) | 1.18 | 0.76 to 1.83 | 0.462 |
| **Moderate PRS** |  |  |  |  |  |  |  |  |  |
| No ACEs | 211 | -0.18(0.90) | -0.07 | -0.25 to 0.12 | 0.473 | 42(19.9) | 0.98 | 0.60 to 1.58 | 0.930 |
| ACEs | 309 | 0.12(1.03) | 0.14 | -0.03 to 0.31 | 0.099 | 87(28.2) | 1.40 | 0.92 to 2.16 | 0.121 |
| **High PRS** |  |  |  |  |  |  |  |  |  |
| No ACEs | 176 | -0.18(0.90) | -0.05 | -0.24 to 0.14 | 0.595 | 38(21.6) | 1.10 | 0.67 to 1.81 | 0.712 |
| ACEs | 328 | 0.15(1.05) | 0.20 | 0.03 to 0.36 | **0.022** | 92(28.0) | 1.47 | 0.97 to 2.25 | 0.076 |
| ***P* for interaction** |  |  |  |  | 0.182 |  |  |  | 0.816 |

**Note:** PRS was standardised using z-scores, then categorized into low(<33rd), moderate(33rd-67th) and high (>67th) genetic risk groups. Premenstrual symptoms were standardised using z-score. Low psychological resilience was defined as the lowest quintile of CD-RISC scale. Age, school, age at menarche, alcohol consumption, resilience, ACEs, BMI, depression probable cases, anxiety probable cases and first ten principal components of ancestry were adjusted. *P* for interaction was tested using a likelihood ratio test, comparing the full model (with interaction term) to the reduced model (without interaction term).

## Table S8. Joint associations of PRS for depression and psychosocial and behavioural factor score with premenstrual symptoms and probable PMDs, additionally adjusted for depression and anxiety

| **Subgroup** | ***N*** | **Premenstrual symptoms** | | | **Probable PMDs** | | |
| --- | --- | --- | --- | --- | --- | --- | --- |
|  |  | **z-score, mean (SD)** | ***β* (95%CI)** | ***P* value^1^** | **N (%)** | ***OR* (95%CI)** | ***P* value^2^** |
| **Low PRS** |  |  |  |  |  |  |  |
| PBS=0 | 135 | -0.17(0.97) | Ref |  | 28(20.7) | Ref |  |
| PBS=1 | 244 | -0.09(0.92) | 0.08(-0.12, 0.29) | 0.414 | 41(16.8) | 0.77(0.45, 1.34) | 0.354 |
| PBS=2-3 | 125 | 0.21(1.14) | 0.27(0.03, 0.50) | **0.026** | 44(35.2) | 1.86(1.06, 3.32) | **0.032** |
| **Moderate PRS** |  |  |  |  |  |  |  |
| PBS=0 | 136 | -0.22(0.90) | -0.02(-0.25, 0.21) | 0.883 | 25(18.4) | 0.87(0.47, 1.60) | 0.653 |
| PBS=1 | 227 | 0.01(0.93) | 0.18(-0.02, 0.39) | 0.082 | 57(25.1) | 1.25(0.74, 2.13) | 0.410 |
| PBS=2-3 | 157 | 0.18(1.11) | 0.25(0.03, 0.48) | **0.026** | 47(29.9) | 1.45(0.84, 2.56) | 0.188 |
| **High PRS** |  |  |  |  |  |  |  |
| PBS=0 | 113 | -0.34(0.78) | -0.11(-0.35, 0.14) | 0.390 | 20(17.7) | 0.85(0.44, 1.62) | 0.623 |
| PBS=1 | 226 | -0.01(0.92) | 0.18(-0.03, 0.39) | 0.086 | 59(26.1) | 1.38(0.82, 2.35) | 0.228 |
| PBS=2-3 | 165 | 0.36(1.16) | 0.45(0.23, 0.68) | **<0.001** | 51(30.9) | 1.59(0.92, 2.77) | 0.100 |
| ***P* for interaction** |  | 0.269 | | | 0.128 | | |

**Note:** PBS (psychosocial and behavioural factor score): participants scored 1 point if they had alcohol consumption, low resilience or exposure to ACEs. PRS was standardised using z-scores, then categorized into low(<33rd), moderate(33rd-67th) and high (>67th) genetic risk groups. Premenstrual symptoms were standardized using z-score. Low psychological resilience was defined as the lowest quintile of CD-RISC scale. Age, school, age at menarche, BMI, depression probable cases, anxiety probable cases and the first ten principal components of ancestry were adjusted. *P* for interaction was tested using a likelihood ratio test, comparing the full model (with interaction term) to the reduced model (without interaction term).

## Table S9. Associations of PRS for depression and psychosocial and behavioural factors with premenstrual affective and physical symptoms

| **Variables** | ***N*** | **Affective symptoms** | | | **Physical symptoms** | | |
| --- | --- | --- | --- | --- | --- | --- | --- |
|  |  | **z-score, mean (SD)** | ***β*_1_(95%CI)** | ***P* value^1^** | **z-score, mean (SD)** | ***β*_2_(95%CI)** | ***P* value^2^** |
| **PRS of depression^a^** |  |  |  |  |  |  |  |
| Low PRS | 504 | -0.06(0.98) | Ref |  | -0.02(1.01) | Ref |  |
| Moderate PRS | 520 | 0.01(1.00) | 0.09(-0.03, 0.22) | 0.135 | 0.00(0.97) | 0.05(-0.07, 0.18) | 0.390 |
| High PRS | 504 | 0.05(1.02) | 0.14(0.01, 0.26) | **0.030** | 0.03(1.02) | 0.10(-0.02, 0.23) | 0.105 |
| *P* for trend |  |  |  | **0.030** |  |  | 0.105 |
| **Alcohol consumption^b^** |  |  |  |  |  |  |  |
| No | 1099 | -0.06(0.96) | Ref |  | -0.05(0.96) | Ref |  |
| Yes^d^ | 429 | 0.16(1.09) | 0.20(0.09, 0.31) | **<0.001** | 0.14 (1.08) | 0.17(0.06, 0.28) | **0.002** |
| **Psychological resilience^b^** |  |  |  |  |  |  |  |
| **Quintiles** |  |  |  |  |  |  |  |
| Q5 | 286 | -0.11(0.99) | Ref |  | -0.15(0.99) | Ref |  |
| Q4 | 311 | -0.08(0.98) | 0.00(-0.16, 0.16) | 0.980 | -0.04(0.94) | 0.09(-0.06, 0.25) | 0.247 |
| Q3 | 318 | -0.11(0.88) | -0.02(-0.18, 0.14) | 0.793 | -0.07(0.92) | 0.06(-0.09, 0.22) | 0.427 |
| Q2 | 304 | 0.01(0.96) | 0.10(-0.06, 0.26) | 0.225 | 0.03(0.97) | 0.16(-0.00, 0.32) | 0.055 |
| Q1 | 309 | 0.29(1.13) | 0.36(0.20, 0.52) | **<0.001** | 0.22(1.13) | 0.33(0.17, 0.49) | **<0.001** |
| **Binary** |  |  |  |  |  |  |  |
| High | 1219 | -0.07(0.95) | Ref |  | -0.06(0.96) | Ref |  |
| Low^e^ | 309 | 0.29(1.13) | 0.34(0.22, 0.46) | **<0.001** | 0.22(1.13) | 0.25(0.13, 0.37) | **<0.001** |
| **ACEs^b^** |  |  |  |  |  |  |  |
| No | 605 | -0.12(0.93) | Ref |  | -0.14(0.95) | Ref |  |
| Yes | 923 | 0.08(1.04) | 0.16(0.06, 0.26) | **0.002** | 0.09(1.02) | 0.20(0.10, 0.30) | **<0.001** |
| **BMI (kg/m^2^)^b^** |  |  |  |  |  |  |  |
| <18.5 | 335 | 0.07(1.02) | 0.10(-0.02, 0.22) | 0.095 | 0.06(1.01) | 0.10(-0.03, 0.21) | 0.142 |
| 18.5-25 | 1108 | -0.02(1.00) | Ref |  | -0.02(1.00) | Ref |  |
| ≥25 | 85 | 0.02(0.96) | 0.00(-0.22, 0.22) | 0.986 | -0.02(0.90) | -0.01(-0.25, 0.18) | 0.759 |
| **PBS^c^** |  |  |  |  |  |  |  |
| 0 | 384 | -0.22(0.86) | Ref |  | -0.23(0.91) | Ref |  |
| 1 | 697 | -0.05(0.93) | 0.16(0.03, 0.28) | **0.013** | -0.01(0.94) | 0.21(0.09, 0.34) | **0.001** |
| 2-3 | 447 | 0.27(1.14) | 0.48(0.34, 0.61) | **<0.001** | 0.22(1.12) | 0.45(0.31, 0.58) | **<0.001** |
| *P* for trend |  |  |  | **<0.001** |  |  | **<0.001** |

**Note: a**: Age and the first ten principal components of ancestry were adjusted. **b**: Age, school, age at menarche, alcohol consumption, resilience, ACEs and BMI were adjusted. **c**: PBS (psychosocial and behavioural factor score); Age, school and age at menarche were adjusted. **d**: Alcohol consumption was defined as consuming alcohol at least once during the past 30 days. **e:** Low psychological resilience: in the lowest quintile of the CD-RISC score

## Table S10. Joint associations of PRS for depression and psychosocial and behavioural factors with premenstrual affective/physical symptoms

| **Variables** | ***N*** | **Affective symptoms** | | | **Physical symptoms** | | |
| --- | --- | --- | --- | --- | --- | --- | --- |
|  |  | **z-score, mean (SD)** | ***β*_1_(95%CI)** | ***P* value^1^** | **z-score, mean (SD)** | ***β*_2_(95%CI)** | ***P* value^2^** |
| **Low PRS** |  |  |  |  |  |  |  |
| No alcohol consumption | 373 | -0.12(0.92) | Ref |  | -0.07(0.97) | Ref |  |
| Alcohol consumption | 131 | 0.11(1.12) | 0.22(0.02, 0.42) | **0.030** | 0.12(1.11) | 0.19(-0.01, 0.38) | 0.064 |
| **Moderate PRS** |  |  |  |  |  |  |  |
| No alcohol consumption | 370 | -0.05(0.97) | 0.08(-0.06, 0.23) | 0.286 | -0.02(0.98) | 0.08(-0.06, 0.23) | 0.253 |
| Alcohol consumption | 150 | 0.16(1.06) | 0.27(0.09, 0.46) | **0.005** | 0.04(0.96) | 0.11(-0.07, 0.30) | 0.234 |
| **High PRS** |  |  |  |  |  |  |  |
| No alcohol consumption | 356 | -0.01(0.98) | 0.13(-0.02, 0.28) | 0.092 | -0.07(0.94) | 0.05(-0.10, 0.19) | 0.534 |
| Alcohol consumption | 148 | 0.19(1.10) | 0.29(0.10, 0.48) | **0.002** | 0.26(1.15) | 0.32(0.13, 0.51) | **0.001** |
| ***P* for interaction** |  |  |  | 0.915 |  |  | 0.194 |
| **Low PRS** |  |  |  |  |  |  |  |
| High psychological resilience | 410 | -0.13(0.93) | Ref |  | -0.08(0.98) | Ref |  |
| Low psychological resilience | 94 | 0.25 (1.12) | 0.36(0.14, 0.58) | **0.001** | 0.23(1.09) | 0.29(0.07, 0.51) | **0.009** |
| **Moderate PRS** |  |  |  |  |  |  |  |
| High psychological resilience | 410 | -0.02(0.98) | 0.12(-0.02, 0.25) | 0.087 | -0.03(0.93) | 0.08(-0.06, 0.21) | 0.265 |
| Low psychological resilience | 110 | 0.12(1.09) | 0.24(0.04, 0.45) | **0.022** | 0.08(1.12) | 0.17(-0.04, 0.37) | 0.114 |
| **High PRS** |  |  |  |  |  |  |  |
| High psychological resilience | 399 | -0.07(0.94) | 0.07(-0.07, 0.21) | 0.335 | -0.06 (0.95) | 0.05(-0.09, 0.18) | 0.494 |
| Low psychological resilience | 105 | 0.51(1.17) | 0.63(0.42, 0.85) | **<0.001** | 0.37 (1.18) | 0.47(0.25, 0.68) | **<0.001** |
| ***P* for interaction** |  |  |  | **0.013** |  |  | 0.085 |
| **Low PRS** |  |  |  |  |  |  |  |
| No ACEs | 218 | -0.10(0.94) | Ref |  | -0.06(1.05) | Ref |  |
| ACEs | 286 | -0.02(1.01) | 0.07(-0.11, 0.25) | 0.428 | 0.01(0.98) | 0.05(-0.13, 0.22) | 0.588 |
| **Moderate PRS** |  |  |  |  |  |  |  |
| No ACEs | 211 | -0.17(0.89) | -0.04(-0.23, 0.15) | 0.673 | -0.16(0.91) | -0.07(-0.25, 0.12) | 0.497 |
| ACEs | 309 | 0.14(1.06) | 0.23(0.06, 0.41) | **0.009** | 0.10(1.00) | 0.17(-0.01, 034) | 0.059 |
| **High PRS** |  |  |  |  |  |  |  |
| No ACEs | 176 | -0.09(0.97) | 0.05(-0.15, 0.24) | 0.658 | -0.22(0.86) | -0.12(-0.31, 0.08) | 0.244 |
| ACEs | 328 | 0.12(1.03) | 0.23(0.06, 0.41) | **0.008** | 0.16(1.07) | 0.25(0.08, 0.42) | **0.005** |
| ***P* for interaction** |  |  |  | 0.268 |  |  | **0.065** |

**Note:** The PRS was standardised using z-score, then categorized into low(<33rd), moderate(33rd-67th) and high (>67th) genetic risk groups. Linear regression model was used to estimate the joint associations of the PRS for depression and psychosocial and behavioural factors with affective and physical symptoms. Low psychological resilience was defined as the lowest quintile of CD-RISC scale. Age, school, age at menarche, alcohol consumption, resilience, ACEs, BMI and first ten principal components of ancestry were adjusted. *P* for interaction was tested using a likelihood ratio test, comparing the full model (with interaction term) to the reduced model (without interaction term).

## Table S11. Joint associations of PRS for depression and psychosocial and behavioural factor score with premenstrual affective/physical symptoms

| **Subgroup** | ***N*** | **Affective symptoms** | | | **Physical symptoms** | | |
| --- | --- | --- | --- | --- | --- | --- | --- |
|  |  | **z-score, mean (SD)** | ***β*_1_ (95%CI)** | ***P* value^1^** | **z-score, mean (SD)** | ***β*_2_ (95%CI)** | ***P* value^2^** |
| **Low PRS** |  |  |  |  |  |  |  |
| PBS=0 | 135 | -0.19(0.86) | Ref |  | -0.15(1.03) | Ref |  |
| PBS=1 | 244 | -0.12(0.91) | 0.07(-0.13, 0.28) | 0.481 | -0.06(0.95) | 0.11(-0.09, 0.32) | 0.275 |
| PBS=2-3 | 125 | 0.21(1.17) | 0.39(0.15, 0.63) | **0.001** | 0.19(1.08) | 0.33(0.09, 0.57) | **0.006** |
| **Moderate PRS** |  |  |  |  |  |  |  |
| PBS=0 | 136 | -0.21(0.88) | 0.00(-0.23, 0.24) | 0.988 | -0.21(0.90) | -0.01(-0.25, 0.22) | 0.917 |
| PBS=1 | 227 | -0.03(0.95) | 0.19(-0.02, 0.40) | 0.082 | 0.04(0.93) | 0.23(0.02, 0.44) | **0.028** |
| PBS=2-3 | 157 | 0.25(1.13) | 0.46(0.23, 0.69) | **<0.001** | 0.11(1.07) | 0.30(0.07, 0.52) | **0.010** |
| **High PRS** |  |  |  |  |  |  |  |
| PBS=0 | 113 | -0.26(0.84) | -0.03(-0.28, 0.22) | 0.813 | -0.36(0.76) | -0.14(-0.39, 0.10) | 0.250 |
| PBS=1 | 226 | -0.01(0.95) | 0.22(0.00, 0.43) | **0.046** | -0.02(0.93) | 0.20(-0.01, 0.41) | 0.068 |
| PBS=2-3 | 165 | 0.33(1.15) | 0.55(0.32, 0.78) | **<0.001** | 0.35(1.17) | 0.56(0.34, 0.78) | **<0.001** |
| ***P* for interaction** |  | 0.783 | | | 0.066 | | |

**Note: PBS** (psychosocial and behavioural factor score): participants scored 1 point if they had alcohol consumption, low resilience or exposure to ACEs. PRS was standardised using z-scores, then categorized into low(<33rd), moderate(33rd-67th) and high (>67th) genetic risk groups. Premenstrual affective/physical symptoms were standardized using z-score. Low psychological resilience was defined as the lowest quintile of CD-RISC scale. Age, school, age at menarche, BMI, and the first ten principal components of ancestry were adjusted. ***P* for interaction** was tested using a likelihood ratio test (LRT), comparing the full model (with interaction term) to the reduced model (without interaction term).

## Table S12. Comparison of characteristics between included and excluded participants

| **Characteristics** | **Included participants N(%)** | **Excluded participants N(%)** | ***P* value** |
| --- | --- | --- | --- |
| Total participants | 1528 | 405 |  |
| Age (years) | 20.05±1.47 | 24.45±1.99 | **<0.001** |
| Academic school |  |  | **<0.001** |
| Clinical Medicine | 1183(77.4) | 86(21.2) |  |
| Other | 345(22.6) | 319(78.8) |  |
| Age at menarche (years) | 12.34±1.23 | 12.45±1.21 | 0.098 |
| Depression |  |  |  |
| No | 1401(91.7) | 377(93.1) | 0.357 |
| Yes | 127(8.3) | 28(6.9) |  |
| Anxiety |  |  |  |
| No | 1390(91.0) | 366(90.4) | 0.711 |
| Yes | 138(9.0) | 39(9.6) |  |
| PMDs |  |  |  |
| No | 1156(75.7) | 297(73.3) | 0.336 |
| Yes | 372(24.3) | 108(26.7) |  |


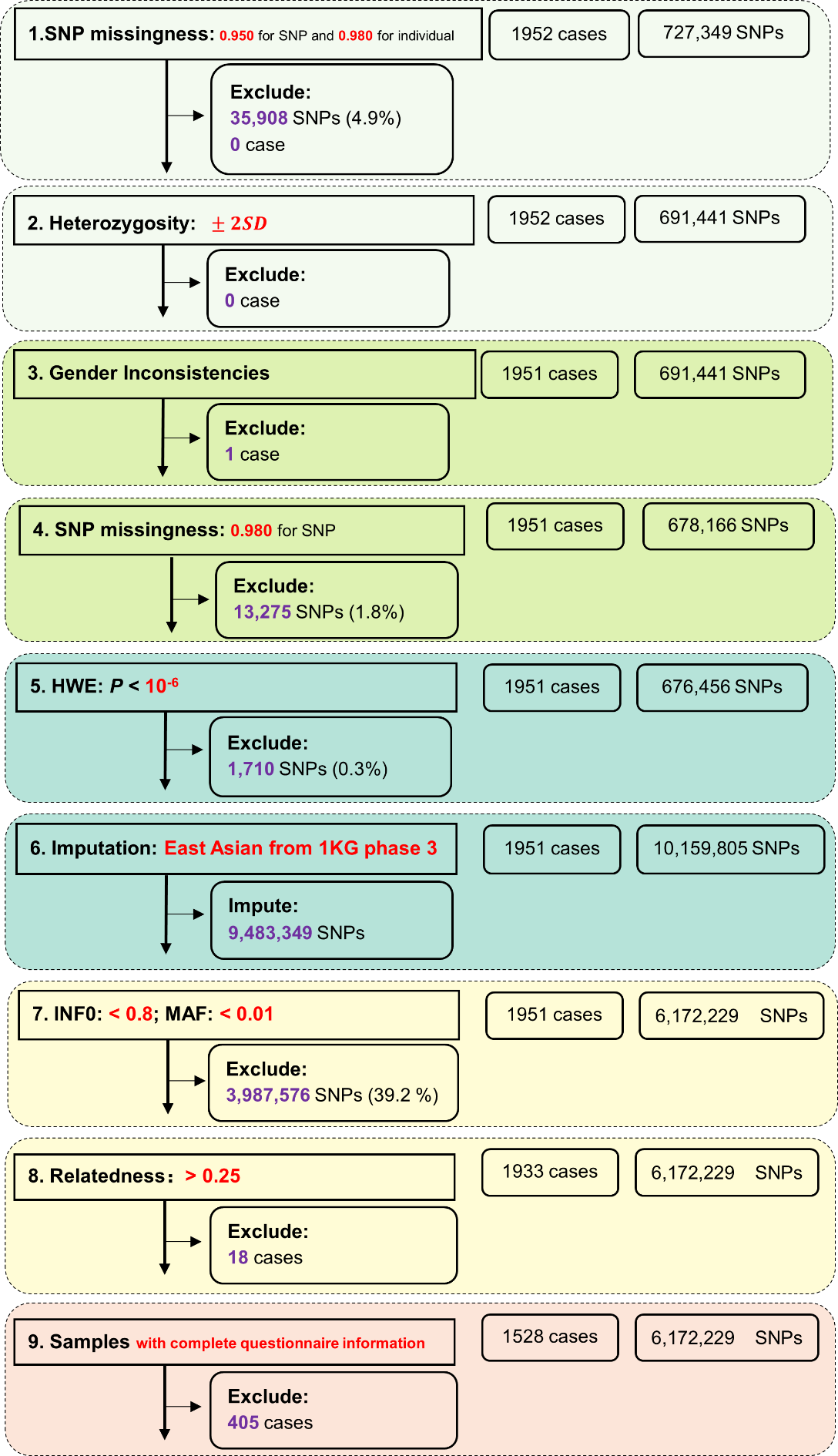


## Figure S1. The quality control and imputation procedure of COPE
